# Perceived Barriers to Providing Spiritual Care in Palliative Care among Professionals: A Portuguese Cross-Sectional Study

**DOI:** 10.3390/ijerph20126121

**Published:** 2023-06-14

**Authors:** Carlos Laranjeira, Maria Anjos Dixe, Ana Querido

**Affiliations:** 1 School of Health Sciences, Polytechnic of Leiria, Campus 2, Morro do Lena, Alto do Vieiro, Apartado 4137, 2411-901 Leiria, Portugal; maria.dixe@ipleiria.pt (M.A.D.); ana.querido@ipleiria.pt (A.Q.); 2 Centre for Innovative Care and Health Technology (ciTechCare), Polytechnic of Leiria, Rua de Santo André—66–68, Campus 5, 2410-541 Leiria, Portugal; 3 Comprehensive Health Research Centre (CHRC), University of Évora, 7000-801 Évora, Portugal; 4 Group Innovation & Development in Nursing (NursID), Center for Health Technology and Services Research (CINTESIS@RISE), 4200-450 Porto, Portugal

**Keywords:** palliative care competence, spirituality, spiritual care, barriers, intervention, Portugal

## Abstract

Spiritual care is an important dimension of palliative care (PC) and a facet of holistic care that helps ill people find meaning in their suffering and lives. This study aims to: (a) develop and test the psychometric properties of a new instrument, Perceived Barriers to Spiritual Care (PBSC); (b) explore participants’ perceptions of how prevalent those (pre-identified) barriers are; and (c) examine the association of their personal and professional characteristics with those perceptions. A descriptive cross-sectional study was carried out using a self-reporting online survey. In total, 251 professionals registered with the Portuguese Association of Palliative Care (APCP) completed the study. The majority of respondents were female (83.3%), nurses (45.4%), had more than 11 years of professional experience (66.1%), did not work in PC (61.8%), and had a religious affiliation (81.7%). The psychometric assessment using PBSC provided sound evidence for its validity and reliability. The three most common perceived barriers were late referral for palliative care (78.1%), work overload (75.3%), and uncontrolled physical symptoms (72.5%). The least commonly perceived barriers were different spiritual beliefs among professionals (10.8%), differences between the beliefs of professionals and patients (14.4%), and the shame of approaching spirituality in a professional context (26.7%). The findings show there is some relationship between sex, age, years of professional experience, working in PC, having a religious affiliation, the importance of spiritual/religious beliefs, and responses to the PBSC tool. The results highlight the importance of advanced training in spirituality and intervention strategies. Further research is needed to properly study the impacts of spiritual care and establish outcome assessments that accurately reflect the effects of the various spiritual care activities.

## 1. Introduction

Professionals use palliative care skills and practices to satisfy the needs of people with life-threatening illnesses, regardless of prognosis [1]. Palliative care (PC) stresses the prompt identification and meeting of medical, psychological, spiritual, or existential needs, as well as the care of families [2,3]. The prime focus of PC is to promote and improve the quality of life for patients and their families throughout the disease’s progression, including maximizing dignity in dying, acknowledging patient choice and autonomy, and addressing the needs of both patients and families in any care environment [4].

Spirituality is a central and historical aspect of PC, assuming significance amid other areas of health. Empirical research reports positive effects of spirituality in PC, but the evidence for the effects of spiritual care is low [5]. Modern scientific healthcare technology will inevitably recover the old consideration of spiritual care. By recognizing the importance and relevance of spiritual needs to a person’s overall well-being, we can meet the holistic care paradigm [6], approaching the person as a whole, including their deepest aspect: the spirit.

According to Nolan et al. [7], spirituality is a dynamic dimension of existence that pertains to how patients express themselves and/or seek meaning, purpose in life, and transcendence. One of PC’s fundamental aims is meeting the spiritual needs of patients [5]. Spirituality is a means to connect to oneself, others, nature, meaning, and/or the sacred during a particular moment [7,8]. Spirituality also encompasses the transcendent dimension of believing in a higher entity and is associated with more humanistic and person-centered care [9]. Patients who reported their spiritual needs were strongly supported by their medical team (e.g., physicians, nurses, and chaplains) and had greater QoL at the end of life [3,10]. Similarly, ill people whose spiritual needs were strongly supported received better hospice care and less aggressive measures at the end of life (e.g., ventilation and resuscitation) [3,11].

Other research has shown that inquiring about a patient’s spiritual concerns has plenty of benefits. Through non-medical discussion, such inquiry allows physicians to get to know patients better [12] and enhances doctor-patient interactions by strengthening trust [13]. A patient’s ability to manage and find meaning in their illness experience increases when they feel acknowledged and validated by healthcare staff [14].

Spiritual care also helps the patient discover their identity and self-knowledge; promotes relationships with others; intensifies contact with nature; reduces anxiety levels; produces comfort and provides well-being and inner peace; promotes a relationship with a higher deity; helps the person feel comforted; fights fear and loneliness; maintains inner strength; and promotes hope. In this way, it can be said that spiritual care is individualized and singular [6,15,16].

The provision of spiritual care as part of healthcare services is gaining popularity but is sub-optimal within and across palliative care services internationally [8]. Spiritual care guidelines for PC patients have been developed and frequently recommend evaluation of spiritual issues using validated instruments (allowing identification of a patient’s hopes, dreams, fears, and beliefs), incorporation of their spiritual concerns into their everyday care, and performance of life completion activities (such as a life review). Indeed, this care is best delivered by well-educated, multidisciplinary, and especially pastoral care workers [17].

Few studies have focused explicitly on PC and the provision of spiritual/existential care, but the scant research suggests that (1) healthcare professionals (HCP) see this care as a component of their holistic care role [9]; (2) there are inherent difficulties in providing spiritual care, such as lack of time and difficulty documenting spiritual care needs [18]; and (3) HCP-patient relationships, therapeutic touch, active listening, communication, being spiritually self-aware, being present, and co-creating fluid care are vital elements [19]. Furthermore, (4) perceived barriers to spiritual care include external or organizational barriers linked to the environment, education, and organization of care, and individual barriers associated with personal beliefs, motivation, and communication [20].

Although one of PC’s basic competencies is to satisfy the spiritual needs of patients with life-threatening illnesses, research has shown that professionals frequently neglect to include spiritual care in care plans [18,20]. This could be attributed to a perception that professionals lack the necessary abilities to provide spiritual assistance to patients on an individual basis, a dearth of spiritual education in training programs, and a shortage of time that gives the impression that spiritual care is being neglected [8,15,20]. Relevant organizational hurdles to delivering spiritual care include noncompliance with workforce standards and managers’ disregard for the significance of providing holistic care [20]. In the Portuguese context, there is currently a dearth of information regarding the barriers to spiritual care faced by professionals in palliative care. We developed a new instrument, the Perceived Barriers to Spiritual Care (PBSC), to measure the extent of these barriers. No valid, reliable, and culturally specific instrument exists in Portuguese that is appropriate for measuring spiritual care barriers in palliative care.

Therefore, the aims of this study were: (1) to develop and test the psychometric properties of a new instrument, Perceived Barriers to Spiritual Care (PBSC); (2) to explore participants’ perceptions of how prevalent those (pre-identified) barriers are; and (3) to examine the association of their personal and professional characteristics with those perceptions.

## 2. Materials and Methods

### 2.1. Study Design

An online descriptive cross-sectional survey was conducted over two months in 2021. In this study, we adhered to the STROBE (Strengthening the Reporting of Observational Studies in Epidemiology) checklist [21].

### 2.2. Sample Selection and Recruitment

A convenience sampling method was applied. Inclusion criteria for the study included: (1) professionals registered in the Portuguese Association of Palliative Care (APCP); (2) being able to communicate in Portuguese; and (3) being willing to participate in this study. Participants who did not meet the inclusion criteria were excluded.

Professionals were invited to participate by several means: the link to the survey was sent via the APCP mailing list and posted on the APCP home page for one month. IP filtering was used to avoid duplicate and repeatedly submitted responses from a single system.

In reliability and validity studies, a sample size ten times the number of items is considered sufficient [22]. As the scale consists of 12 items, a sample of at least 120 individuals was considered sufficient for the study. Out of 414 registered professionals, 251 accepted to participate and were enrolled (a response rate of 60.6%).

### 2.3. Instruments

The e-survey instrument included two parts:

**(1) Personal and professional information**, including age, sex, profession, years of professional experience, working in palliative care (Yes/No), religious beliefs (Yes/No), religious affiliation (Catholic/Other), and degree of importance of spiritual/religious beliefs measured on a five-point Likert-type scale ranging from 1 (“not at all important”) to 5 (“extremely important”).

**(2) Perceived barriers to spiritual care (PBSC) tool**, the PBSC is a 12-item self-rated instrument designed to measure professionals’ agreement regarding barriers to providing spiritual care for patients. Responses to each item of the PBSC tool are measured on a five-point Likert-type scale ranging from 1 (“strongly disagree”) to 5 (“strongly agree”). The total score of the PBSC tool is calculated by summing the responses from all 12 items. The range of scores is 12 to 60, with higher scores indicating greater perceived barriers to spiritual care.

The process of instrument development and validation followed four phases. In phase 1, 12 items were generated from the literature review [23,24]. In phase 2, the content validity of the preliminary items was assessed and reviewed by a focus group of twelve experts in academia, instrument development research, and palliative care (two nurses, two physicians, two social workers, two physiotherapists, two psychologists, and two spiritual assistants). This focus group revised the items for comprehensibility and language, cultural, and spiritual consistency to suit the Portuguese population. Interrater reliability was determined using the percentage of agreement [25], and a consensus was considered if an agreement was equal to or greater than 90%. Two items were revised based on the experts’ comments, and all 12 items were retained for construct validation. In phase 3, pretesting the preliminary version of the PBSC on a convenience sample of 15 professionals in palliative care to ensure clarity, familiarity, and suitability of the words and phrases used. Their comments revealed that the items were not confusing, were suitable, and were easy to complete. Lastly, phase 4 tested the final version of the PBSC by evaluating its internal structure using internal consistency reliability (Cronbach’s alpha) and exploratory factor analysis (EFA).

### 2.4. Ethics

This study complied with all ethical guidelines stated in the Helsinki Declaration. The Local Ethics Committee provided approval before the study was conducted (study protocol n. CE-IPLEIRIA-02-2019). Before performing the survey, participants were given a brief explanation of its purpose and goals and confirmed their willingness to continue by checking a box. All responses were handled discreetly and anonymously. All respondents’ identities and anonymity were protected, and all data was maintained securely. Respondents were asked to evaluate a personal aspect they may not have previously thought about, but other than this, there were no real ethical concerns or hazards associated with participation.

### 2.5. Data Analysis

The internal consistency of the PBSC and the proposed factors of the exploratory factor analysis were measured using Cronbach’s alpha. Cronbach’s coefficient of 0.70 was deemed the minimum level for evidence of internal consistency [25]. Results from both Kaiser-Meyer-Olkin (KMO) and Bartlett’s test of sphericity demonstrated that the sample met the factor analysis criteria [25]. A KMO score larger than 0.50 and the significance of Bartlett’s test of sphericity value were used to determine the factorability of the data (*p* < 0.05) [25].

The construct validity of the PBSC was demonstrated by exploratory factor analysis (EFA). The principal components method with varimax rotation was used to extract common factors. A variable can typically be attributed to a factor if the factor loading is >0.4 [26].

The Kolmogorov-Smirnov test was used to determine whether the data followed a normal distribution. Comparisons between quantitative variables (barriers) and qualitative variables (sex, working in palliative care, and religious beliefs) were carried out using the student’s *t*-test. We also performed a Pearson correlation test between the quantitative variables. All variables were used in the analysis except the participants’ profession because the sample size was very small in some categories (1–6 observations) and categories could not be aggregated. The use of this variable could produce a spurious relationship [27]. In the analyses, a *p*-value of less than 0.05 was regarded as statistically significant. SPSS 28.0 software (SPSS Inc., Chicago, IL, USA) was used for all data analysis.

## 3. Results

### 3.1. Sample Description

Descriptive findings were given in totals, percentages, medians, means, and standard deviation values. The sample included 251 professionals who responded to a survey released by the Portuguese Palliative Care Association. Most of the participants (83.3%) were female nurses (45.4%) with an average age of 42.83 ± 11.9 and an average of 16.7 ± 10.4 years of professional activity (Table 1). Although APCP members, 96 participants were not currently exercising their professional activity in palliative care. The average importance of spiritual/religious beliefs (4.06 ± 1.02) was higher than the scale’s median, indicating that participants attributed high importance to such beliefs.

### 3.2. Psychometric Properties of the PBSC

Bartlett’s test of sphericity was significant (χ^2^ = 1120.10, *p* < 0.001), and the KMO measure of sample adequacy was 0.715. A two-factor solution for the PBSC was identified using exploratory factor analysis, with factor 1 representing “Contextual and Assistance-related Barriers” (five items) and factor 2 representing “Intra and Interpersonal-related Barriers” (seven items), conceptual meanings that best match this two-factor solution. Kaiser’s eigenvalue-greater-than-one criterion is supported by the fact that each of these factors has an eigenvalue greater than 1 and accounts for a sizable portion (46.7%) of the variation between items. The two-factor structure matrix with each item’s loadings on each factor is shown in Table 2. This scale’s Cronbach’s alpha value was 0.830, which indicated strong internal consistency [25,26]. Cronbach’s α coefficient of both factor 1 (0.781) and factor 2 (0.741) also indicated satisfactory internal consistency [26].

### 3.3. Perceived Barriers to Spiritual Care Provision

Table 3 depicts the measures of central tendency (item mean and standard deviation) and the frequencies and percentages of responses to all items of the PBSC. Levels of agreement on each scale item showed the most and least common barriers inhibiting the provision of spiritual care. The three most common perceived barriers were late referral for palliative care (78.1%), work overload (75.3%), and dealing with uncontrolled physical symptoms (72.5%). The least commonly perceived barriers were different spiritual beliefs among professionals (10.8%), differences between the beliefs of professionals and patients (14.4%), and the shame of approaching spirituality in a professional context (26.7%).

### 3.4. Associations between PBSC and Sex, Working in Palliative Care, and Having Religious Beliefs

A person’s sex seems to influence how decisions are made [28]. We therefore hypothesized that perceived barriers might vary between the sexes. As Table 4 shows, women had higher mean levels of agreement with barriers. There were significant differences between men and women in factor 2 (“Contextual and Assistance-related Barriers”) of the PBSC and regarding items 3, 7, and 8. Student’s *t*-test also showed that professionals with religious beliefs scored higher in the overall scale and in each factor, and that palliative care practitioners exhibited fewer perceived barriers to spiritual care, except for item 4 (“Late referral for palliative care”), which had a larger mean compared to individuals who do not work in PC (4.24 ± 0.97 vs. 3.93 ± 1.03; *t*-test −2.411, *p* < 0.001).

### 3.5. Correlations between PBSC and Age, Years of Professional Experience, and Having Religious Beliefs

The findings revealed an association between age, years of professional experience, the importance of spiritual/religious beliefs, and the responses to the PBSC instrument (Table 5). High scores on item 2 (“work overload”) were negatively related to age (r = −0.153, *p* < 0.05) and years of professional experience (r = −0.157, *p* < 0.05). Similarly, high scores on item 1 (“lack of time”) were negatively related to years of professional experience (r = −0.124, *p* < 0.05). High scores on item 5 (“fear of revealing matters that could make the situation worse”) were positively related to age, and high scores on item 10 (“differences between the beliefs of professionals and patients”) were positively related to the importance of spiritual/religious beliefs (r = 0.150, *p* < 0.05). Lastly, both PBSC factors were positively related to the importance of spiritual/religious beliefs (factor 1, r = 0.184, *p* < 0.001; factor 2, r = 0.174, *p* < 0.001).

## 4. Discussion

The psychometric evaluation of PBSC was established and proven in this study with a sample of professionals registered with the Portuguese Palliative Care Association. The psychometric evaluation offered strong support for its validity and reliability. Our findings also demonstrated adequate content and reliability [25].

For our participants, the most common barriers to providing spiritual care (with agreement varying between 59.3% and 78.1%) were contextual and assistance-related factors such as lack of privacy, lack of relational and communication skills, lack of competent professionals on the team, lack of time, dealing with uncontrolled physical symptoms, and late referral for PC. These results are aligned with other studies indicating that lack of private space, insufficient time, and lack of competent staff are among the prevalent barriers to providing spiritual care [23,29,30,31]. Professionals identify late referral to PC as the barrier that obtained the highest agreement, frequently resulting in subpar pain and symptom management, increased suffering, and unplanned hospital deaths [32]. This complexity results in patients’ spiritual needs not always being recognized and receiving inappropriate treatment [33].

Overall, studies reveal that end-of-life (EOL) patients have spiritual needs that are not adequately addressed by HCPs. Understanding how HCPs perceive the importance of spiritual care, as well as the difficulties and barriers that they face in the performance of their profession, is increasingly important. Our results agree with those of Arrieira et al. [34], who performed phenomenological interviews with professionals working in PC. These authors verified that professionals refer to a lack of training in how to approach spirituality, resulting in the denial of spiritual care. They add that health courses are poor in terms of EOL curricula, which in effect leads to the dehumanization of care.

In the study by Canfield et al. [35], nurses asked to identify barriers to providing spiritual care indicated issues related to finitude, resolutions associated with guilt and hope, and increased needs for attention. Richardson [36] adds that HCPs often avoid approaching spirituality by shortening interactions with the patient, and they raise concerns about the preservation of privacy and the impossibility of ensuring continuity of care. Failure to address spiritual issues can frustrate attempts to treat other symptoms and adversely affect the patient’s perceived quality of life.

In our study, the least common perceived barriers to providing spiritual care were those related to intrapersonal and interpersonal barriers, with percentages ranging from 10.8% to 43.1%, mainly differing spiritual beliefs among professionals and between professionals and patients; the shame of approaching spirituality in a professional context; spiritual care being taboo for the team; and fear of revealing matters that could worsen the situation. The literature indicates that language and misunderstandings of spiritual care hinder spiritual support [37], but in the present study, these were the least common barriers. Our research highlights the various levels of misunderstanding by demonstrating how assumptions and biases prevent professionals from considering the broad applicability of spiritual care and from clearly communicating the potential benefits of spiritual care to patients and family members [37]. These factors include a lack of familiarity with what spiritual health practitioners do and a limited understanding of when spiritual support is required [37,38]. In Oxhandler and Giardina’s [39] study of social workers, participants had trouble meeting patients’ spiritual needs because they viewed doing so as taboo or falling within the patient’s most private sphere.

The literature also mentions other barriers related to personal and cultural identity. Professionals reveal difficulties in providing care when the patients at the EOL and their families differ culturally from the professional staff [40]. HCPs seem to underuse the standardized models of approaching spirituality, maintaining a highly pharmacological intervention [40]. Richardson [36] highlights the need for a culture of care for EOL patients, as it has an important influence on understanding disease and finitude, adding that HCPs must be increasingly culturally sensitive and competent. Although HCPs recognize spirituality as a facilitator in the face of finitude and an important care tool, some HCPs find that providing spiritual care often presents difficulties due to discomfort and a lack of knowledge and tend to direct their practice more towards biological needs [34].

Our findings show that professionals´ religious convictions affect how they perceive barriers to providing spiritual care. Research by Ronaldson et al. [41] supports this conclusion that people with stronger personal spiritual/religious viewpoints are more likely to conduct spiritual care. Likewise, the effective use of spiritual care can help nurses and doctors find job satisfaction, maintain a positive outlook about their work, and affirm their worth both personally and professionally [42,43].

Western society has become more pluralistic over the last few decades. While institutionalized religion has declined because of secularization, a variety of religious and spiritual viewpoints have emerged in the West as a result of migration, globalization, and the rise of new forms of spirituality [44]. As a result, professional caregivers increasingly care for patients and clients who have different spiritual beliefs than their own, which may occasionally make it more difficult to provide spiritual care [45].

Participants’ perceived barriers to spiritual care were also analyzed according to their age, sex, and years of clinical experience. We found sex differences in some barriers, with women scoring higher than men in items related to a lack of relational and communication skills, a lack of a competent team, and struggles with uncontrolled physical symptoms. These results might be explained because some studies show that women have a deeper connection with the world around them, appear to be more concerned about the emotional well-being of those under their care, and attribute greater value to sharing feelings and communicating about troubling issues [46]. While caring remains a predominantly feminine activity, several authors have highlighted women’s higher emotional and social connectedness towards their patients and their sense of family obligation compared to men [47]. Indeed, it is assumed that women have naturally higher empathy, sensitivity, and willingness to put others before themselves [48].

Furthermore, it appears that older professionals with more clinical expertise understood spirituality and spiritual care better than younger professionals with less experience. This was attributed to the chance to get the knowledge necessary to handle patient concerns [49,50].

Another element that may affect how well-informed clinicians are about the obstacles to providing spiritual care is the clinical setting. The hurdles to spiritual care are lower for professionals in palliative care than for others. This may be explained by the increased use of therapeutic and communication skills in this setting, as well as the higher level of training in spiritual care [40,50,51].

Most participants claimed to be religious (mainly Catholic) and emphasized the importance of spiritual/religious beliefs. People in Portugal generally perceive religion as a fundamental part of life, and this was reflected among the study’s participants. Those professionals who reported higher levels of importance for spiritual/religious beliefs had higher perceived barriers to spiritual care. This significant association seems consistent with the results of Balboni et al. [23]. Spiritual awareness seems to enhance the attention given to what limits the implementation of spiritual care. Furthermore, professionals who value personal spirituality and therapeutic use of themselves can deliver more holistic care and surpass barriers to spiritual care [52].

### 4.1. Study Limitations

This study has some limitations. The generalizability of the results may be constrained by convenience sampling and can be tested in further research with a random sampling technique and a more representative sample from various religious and spiritual traditions. Due to this study’s cross-sectional methodology, we did not examine test-retest reliability, which analyzes the PBSC tool’s temporal stability over time [25]. Nonetheless, additional research is advised to confirm the instrument’s stability and discriminant validity. Furthermore, there will always be certain shortcomings in any attempt to quantify a highly subjective notion like perceived barriers to spiritual care. There is always a chance of bias when using self-reported measurements. Future research should test regression analysis in order to forecast a predictive model. Study participants may respond in a socially acceptable manner. Finally, even though spirituality and religiosity are separate concepts, most participants in our sample were Catholics. Future research spanning different cultures and religions (on a spectrum from highly religious to completely secular) could therefore shed light on the connection between spirituality and spiritual care.

### 4.2. Study Implications

There is a need for more research on how spirituality, spiritual health, and spiritual care are incorporated into academic curricula and theoretical and practical courses. Given its positive effects on holistic and humanized care, training in spiritual care is relevant for delivering sufficient spiritual care [43,53]. By focusing on the inner lives and spirituality of students, educators may be able to use more holistic techniques and include them in a broader strategy for providing spiritual care and exercising hope in palliative care [54,55]. Professionals may find training in spiritual care useful as a means to lower stress at work and enhance the office environment.

During clinical placements, professionals and mentors must provide the best possible examples to help students comprehend and improve their spiritual care competence [56]. Spiritual leadership fosters holistic care and cooperation, two factors that encourage spirituality at work and create a clinical environment that is conducive to learning spiritual care. The recommendations are to (1) integrate spiritual care in the intervention of all palliative care patients; (2) include spiritual care education in the basic and advanced training of all interdisciplinary team members; and (3) involve competent professionals in spiritual care [within a multicultural perspective] in the evaluation and care of palliative patients [57,58].

## 5. Conclusions

Our study supports the satisfactory reliability and validity of the PBSC tool. This newly created tool can be useful in identifying perceived barriers to spiritual care in palliative care. The gap between the PC guidelines and the spiritual care provided by professionals will remain unchanged if the evaluation and provision of spiritual care do not become a standard routine for all professionals who provide EOL care. Care teams must include professionals with competencies in spiritual care who are qualified to respond to spiritual problems. We suggest creating models that promote and teach HCPs how to deal with the spiritual dimension in their work by developing communicative skills, empathy, and relationships in conjunction with listening and respect for the dignity of an ill person. For instance, professionals can easily operationalize spiritual care through the following expressions: “What gives your life meaning?” and “Is spirituality important in your life?”.

In sum, HCPs largely recognize the barriers to spiritual care for patients at the EOL, whether due to work overload, lack of time, lack of privacy, lack of preparation, or late referral for PC. Likewise, these barriers seem to be influenced by their personal characteristics. HCPs must be aware of their role in providing spiritual care to EOL patients as well as in developing patient-centered approach strategies. Training should be expanded at the academic level to promote reflection on spirituality and broaden training.

## Figures and Tables

**Table 1:** Sample characteristics (n = 251).

Variables		n	%
Sex	Male	42	16.7
	Female	209	83.3
Age (years) (42.8 ± 11.09)	18–35	74	29.5
	36–45	81	32.3
	46–55	59	23.5
	56–65	32	12.7
	≥66	5	2.0
Profession	Nurse	114	45.4
	Physician	57	22.7
	Psychologist	12	4.8
	Social worker	18	7.2
	Nutritionist	1	0.4
	Physiotherapist	4	1.6
	Occupational therapist	2	0.8
	Speech therapist	6	2.4
	Spiritual assistant	18	7.2
	Missing	19	7.6
Years of professional experience (16.7 ± 10.4)	<1	2	0.8
	1–5	44	17.5
	6–10	39	15.5
	≥11	166	66.1
Works in palliative care	Yes	155	61.8
	No	96	38.2
Has religious beliefs	Yes	205	81.7
	No	46	18.3
Religious affiliation	Catholic	179	87.3
	Other	26	12.7

**Table 2:** Exploratory factor analysis of the PBSC.

Items		Factor 1	Factor 2
5.	Fear of revealing matters that could make the situation worse	0.509	-
9.	Different spiritual beliefs among professionals	0.713	-
10.	Differences between the beliefs of professionals and patients	0.771	-
11.	Shame of approaching spirituality in a professional context	0.749	-
12.	Spiritual care is *taboo* for the team	0.763	-
1.	Lack of time	-	0.644
2.	Work overload	-	0.711
3.	Dealing with uncontrolled physical symptoms	-	0.653
4.	Late referral for palliative care	-	0.586
6.	Lack of privacy	-	0.604
7.	Lack of relational and communication skills	-	0.566
8.	Lack of competent professionals on the team	-	0.445
Eigenvalue		3.912	1.696
Sub-total percentage of variance explained		23.53%	23.19%
Total percentage of the factors explained		46.70%	

**Table 3:** Participants’ agreement on the twelve items of the PBSC (n = 251).

Item	Item (Mean ± SD; Median)	Strongly Disagree		Disagree		Neutral		Agree		Strongly Agree		Agreement	
		n	%	n	%	n	%	n	%	n	%	n	%
1. Lack of time	3.76 ± 1.10; 4	12	4.8	20	8.0	57	22.7	89	35.5	73	29.1	162	64.6
2. Work overload	3.97 ± 1.00; 4	6	2.4	19	7.6	37	14.7	103	41.0	86	34.3	189	75.3
3. Dealing with uncontrolled physical symptoms	3.81 ± 1.03; 4	13	5.2	14	5.6	42	16.7	120	47.8	62	24.7	182	72.5
4. Late referral for palliative care	4.12 ± 1.00; 4	8	3.2	9	3.6	38	15.1	86	34.3	110	43.8	196	78.1
5. Fear of revealing matters that could make the situation worse	3.11 ± 1.16; 3	34	13.5	34	13.5	75	29.9	86	34.3	22	8.8	108	43.1
6. Lack of privacy	3.51 ± 1.11; 4	16	6.4	32	12.7	54	21.5	105	41.8	44	17.5	149	59.3
7. Lack of relational and communication skills	3.78 ± 1.16; 4	13	5.2	24	9.6	52	20.7	78	31.1	84	33.5	162	64.6
8. Lack of competent professionals on the team	3.87 ± 1.13; 4	11	4.4	23	9.2	45	17.9	80	31.9	92	36.7	172	68.6
9. Different spiritual beliefs among professionals	2.02 ± 1.08; 2	110	43.8	56	22.3	58	23.1	23	9.2	4	1.6	27	10.8
10. Differences between the beliefs of professionals and patients	2.14 ± 1.19; 2	103	41.0	58	23.1	54	21.5	24	9.6	12	4.8	36	14.4
11. Shame of approaching spirituality in a professional context	2.45 ± 1.33; 2	88	35.1	48	19.1	48	19.1	49	19.5	18	7.2	67	26.7
12. Spiritual care is taboo for the team	2.61 ± 1.36; 2	72	28.7	58	23.1	45	17.9	49	19.5	27	10.8	76	30.3

**Table 4:** Results of the student *t*-tests for the items of the PBSC scale concerning the variables sex, working in palliative care, and religious beliefs.

	Work in Palliative Care					Having Religious Beliefs					Sex				
	No		Yes		*t*	No		Yes		*t*	Male		Female		*t*
	Mean	SD	Mean	SD		Mean	SD	Mean	SD		Mean	SD	Mean	SD	
Factor 1—Intra and Interpersonal-related Barriers	2.67	0.91	2.34	0.87	2.916 **	2.21	0.84	2.52	0.91	2.184 *	2.55	1.35	2.43	1.33	0.085
5. Fear of revealing matters that could make the situation worse	3.33	1.03	2.97	1.22	2.390 **	3.16	1.16	2.91	1.17	1.282	3.55	1.27	3.51	1.08	−0.968
9. Different spiritual beliefs among professionals	2.17	1.08	1.94	1.08	1.642	2.08	1.11	1.77	0.94	1.811 *	3.36	1.3	3.87	1.11	−1.402
10. Differences between the beliefs of professionals and patients	2.24	1.22	2.08	1.18	1.046	2.22	1.23	1.81	0.95	2.123 *	3.6	1.43	3.93	1.07	0.586
11. Shame of approaching spirituality in a professional context	2.67	1.43	2.31	1.25	2.076 *	2.51	1.359	2.15	1.17	1.703 *	1.81	1.07	2.07	1.09	0.54
12. Spiritual care is taboo for the team	2.96	1.38	2.39	1.306	3.291 **	2.65	1.379	2.4	1.28	1.124	2.24	1.39	2.12	1.15	1.188
Factor 2—Contextual and Assistance-related Barriers	3.93	0.71	3.77	0.65	1.763 *	3.68	0.68	3.87	0.67	1.718 *	2.95	1.15	3.14	1.17	−1.827 *
1. Lack of time	3.88	1.03	3.69	1.13	1.292	3.81	1.09	3.53	1.12	1.585	12.38	4.88	12.32	4.44	0.466
2. Work overload	4.1	0.96	3.89	1.02	1.643	4	0.97	3.83	1.14	1.077	25.62	6.06	27.08	4.41	0.029
3. Dealing with uncontrolled physical symptoms	3.86	0.98	3.78	1.07	0.623	3.84	1.03	3.7	1.06	0.812	3.17	0.77	3.28	0.63	−3.705 **
4. Late referral for palliative care	3.93	1.03	4.24	0.97	−2.411 **	4.14	0.99	4.02	1.07	0.743	3.83	1.21	3.75	1.08	−0.676
6. Lack of privacy	3.7	1.08	3.4	1.12	2.071 *	3.59	1.1	3.19	1.13	2.217 *	3.98	1.09	3.97	0.99	0.214
7. Lack of relational and communication skills in the workforce	3.97	1.12	3.66	1.17	2.030 *	3.82	1.16	3.6	1.13	1.214	3.29	1.07	3.92	1	−2.623 **
8. Lack of competent professionals on the team	4.06	1.06	3.75	1.17	2.095 *	3.87	1.14	3.89	1.1	−0.141	4.02	1.14	4.14	0.98	−1.737 *
Total score of the PBSC scale	40.86	8.09	38.1	7.47	2.756 **	36.81	7.53	39.7	7.8	2.307 *	38	9.2	39.39	7.5	−1.054

* *p* < 0.05; ** *p* < 0.001.

**Table 5:** Correlations between PBSC scores and age, years of professional experience, and having spiritual/religious beliefs.

	Age	Years of Professional Experience	Degree of Importance of Spiritual/Religious Beliefs
Factor 1—Intra and Interpersonal-related Barriers	−0.065	0.008	0.184 **
5. Fear of revealing matters that could make the situation worse	0.134 *	0.109	0.054
9. Different spiritual beliefs among professionals	−0.02	−0.001	0.117
10. Differences between the beliefs of professionals and patients	0.066	−0.015	0.150 *
11. Shame of approaching spirituality in a professional context	0.016	−0.033	0.123
12. Spiritual care is taboo for the team	0.041	−0.035	0.027
Factor 2—Contextual and Assistance-related Barriers	−0.052	0.017	0.174 **
1. Lack of time	−0.108	−0.124 *	0.024
2. Work overload	−0.153 *	−0.157 *	−0.01
3. Dealing with uncontrolled physical symptoms	−0.023	−0.034	−0.041
4. Late referral for palliative care	−0.08	−0.045	−0.116
6. Lack of privacy	0.124	0.099	0.059
7. Lack of relational and communication skills	0.014	0.076	0.082
8. Lack of competent professionals on the team	−0.022	0.008	0.096
Total score of the PBSC scale	0.006	−0.012	0.116

* Correlation is significant at the 0.05 level (two-tailed); ** Correlation is significant at the 0.001 level (two-tailed).

## Data Availability

All data generated or analyzed during this study are included in this article.
